# Review of Whole Head Experimental Cochlear Promontory Vibration with Bone Conduction Stimulation and Investigation of Experimental Setup Effects

**DOI:** 10.1177/23312165211052764

**Published:** 2021-10-28

**Authors:** Srdjan Prodanovic, Stefan Stenfelt

**Affiliations:** 1 Department of Biomedical and Clinical Sciences, 4566 Linköping University, Linköping, Sweden

**Keywords:** bone conduction, LiUHead, finite element model, review

## Abstract

Bone conduction sound transmission in humans has been extensively studied using cochlear promontory vibrations. These studies use vibration data collected from measurements in live humans, whole cadavers, and severed cadaver heads, with stimulation applied either at an implant in the skull bone or directly on the skin. Experimental protocols, methods, and preparation of cadavers or cadaver heads vary among the studies, and it is currently unknown to what extent the aforementioned variables affect the outcome of those studies. The current study has two aims. The first aim is to review and compare available experimental data and assess the effects of the experimental protocol and methods. The second aim is to investigate similarities and differences found between the experimental studies based on simulations in a finite element model, the LiUHead. With implant stimulation, the average cochlear promontory vibration levels were within 10 dB, independent of the experimental setup and preparations of the cadavers or cadaver heads. With on-skin stimulation, the results were consistent between cadaver heads and living humans. Partial or complete replacement of the brain with air does not affect the cochlear promontory vibration, whereas replacing the brain with liquid reduces the vibration level by up to 5 dB. An intact head–neck connection affects the vibration of the head at frequencies below 300–400 Hz with a significant vibration reduction at frequencies below 200 Hz. Removing all soft tissue, brain tissue, and intracranial fluid from the head increases the overall cochlear promontory vibration level by around 5 dB.

Bone conduction (BC) is the phenomenon of sound transmission to the inner ear by vibrations of the head resulting in a hearing sensation, similar to air conduction (AC) hearing by vibrations of the air inside the ear canal (Stenfelt, 2011; Stenfelt & Goode, 2005a). Several different pathways have been recognized and suggested as contributors for BC hearing: ear canal sound pressure, middle ear ossicles inertia, inner ear fluid inertia, compression and expansion of inner ear space, and pressure transmission through the skull interior (Stenfelt, 2012a, 2015; Stenfelt & Goode, 2005a). However, their relative importance is still debated (Stenfelt, 2016, 2020).

A large amount of experimental data used to study BC has been obtained using human cadavers. The use of cadavers is based on the notion that vibration levels in cadavers are similar to those measured in living humans (Eeg-Olofsson et al., 2013). The cochlear promontory vibration is accepted as an indicator of the hearing sensation based on the idea that the vibration at the inner ear dominates the BC hearing in normal ears (Eeg-Olofsson et al., 2008, 2013; Stenfelt & Goode, 2005b; Zhao et al., 2021). However, types of cadavers used for BC experiments varies: dry skulls (i.e., Stenfelt et al., 2000), severed Thiel embalmed heads (i.e., Dobrev et al., 2016), thawed frozen severed heads (i.e., Rigato et al., 2018; Stenfelt & Goode, 2005b) or entire embalmed cadavers (i.e., Eeg-Olofsson et al., 2008). Moreover, BC has been studied using cochlear promontory vibrations in live humans (i.e., Eeg-Olofsson et al., 2013). Because most of the reported measurements of the cochlear promontory vibrations use laser Doppler vibrometry (LDV), they require a free optical pathway and post-mastoidectomy patients or cadaver heads are normally used.

The BC sound transmission depends on the stimulation position (Eeg-Olofsson et al., 2011; Stenfelt, 2012b) as well as on conditions and manipulations of the head and skull (Prodanovic & Stenfelt, 2020; Stenfelt, 2012a; Stenfelt et al., 2002). The different studies of cochlear promontory vibrations differ in methods and preparations that can affect the outcome. In some preparations, the intracranial space was perfused by a cerebrospinal fluid (CSF), thus effectively replacing the brain with liquid (Dobrev et al., 2019). In other experiments, thawed or embalmed cadaver heads’ brain and CSF may have partially leaked out resulting in sections of the cranial interior filled with air. Even if it has been demonstrated that head–neck attachment only affects skull vibrations at low frequencies (Stalnaker & Fogle, 1971) the placement and attachment of the severed heads during the measurements could affect the results.

The primary aim of the current study is to compile and compare cochlear promontory vibration data for BC experimental studies with stimulation in the mastoid area, either at the skin or directly at the skull bone. A secondary aim is to investigate method-related differences based on simulations in a finite element model, the LiUHead.

## Methods

### The Finite Element Model

**Figure 1.**
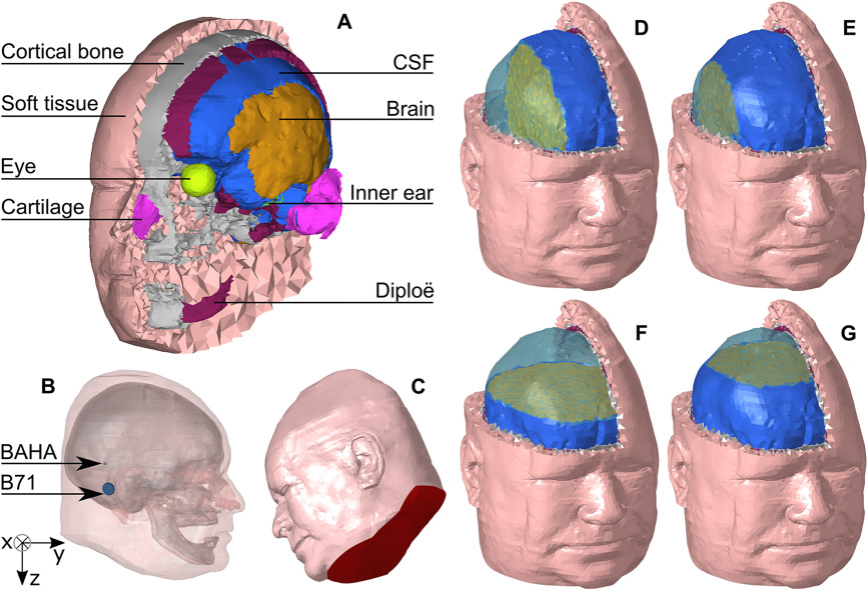
LiUHead Model Overview and Simulation Setup. (A) The layered structure of the LiUHead finite element model where outer layers are removed to reveal inner layers. (B) The stimulation position on the ipsilateral side and the coordinate axes. (C) Red color indicates the part of the head that is constrained in the fixed neck model simulation. The brain and CSF domain manipulation along the medial axis where the ipsilateral hemisphere size is reduced (D) to a width of 1/2 and (E) to a width of 2/3. The brain and CSF domain manipulation along the cranial axis where the brain size is reduced (F) to a height of 1/2 and (G) to a height of 2/3. Semitransparent blue is the air replacing the cavity created by removal of the cranial content. The interface between the brain and added air is colored dull green.

A finite element model, the LiUHead (Figure 1A) (Chang et al., 2016) was used for the simulations. The model comprises approximately 87,000 nodes and 480,000 tetrahedral (4 nodes) elements and contains eight domains: CSF, eyes, inner ear, cortical bone, trabecular bone (diploë), brain, cartilage, and soft tissue (skin, muscles, fat and connecting tissues). The model is solved using COMSOL Multiphysics acoustics and solid mechanics modules (COMSOL Inc., Stockholm, Sweden). Meshing and mesh alterations were carried out using HyperMesh (Altair Engineering, Troy, MI, USA).

CSF = cerebrospinal fluid.

### Simulation Protocol

To investigate different conditions in experiments with human cadaver heads, three types of manipulations of the brain and CSF domains were simulated: (a) reduced volumes of the brain and CSF within the cranial space where the removed tissue is replaced by air, (b) total replacement of the brain and CSF with air (air-brain), and (c) replacement of the brain with CSF-like liquid (liquid-brain). The brain and CSF volumes were reduced separately along the medial axis (from the stimulation side towards the middle of the head or *x*-axis, simulating the case where the head is laid on its side) and cranial axis (top to bottom or *z*-axis, simulating the head standing up). The cerebral hemisphere on the ipsilateral side is reduced to 2/3 or 1/2 of the width along the medial axis (Figure 1D and E), resulting in a brain and CSF volume reduction by 10% and 23%, respectively. Similarly, the cerebrum size is reduced to 2/3 or 1/2 of the height along the cranial axis (Figure 1F and G) resulting in 6% and 33% volume reduction, respectively.

The liquid-brain model was created by assigning mechanical properties of the CSF to the brain, thus creating a single liquid domain within the skull. The air-brain model was created by assigning brain and CSF mechanical properties of air, resulting in an air-filled cranial dome. One special simulation setup, the skull-only model, is created by removing soft tissues and the cartilage from the air-brain model, leaving only bony skull. It is worth noting that the skull-only model simulates a fresh, or living, skull, and it is different from a dry-skull model (Kim et al., 2014; Stenfelt et al., 2000).

In addition to the head manipulations, two boundary conditions for the LiUHead were used: the default boundary conditions (unconstrained outer boundaries) and constrained lower surface of the head—the fixed-neck model (Figure 1B). The fixed-neck model is used to simulate experiments where the cadaver head is attached to the body or clamped at the skull-neck interface. From this point on, the LiUHead standard model refers to the model without head manipulations and with free outer boundaries. Specific manipulations of the model will be stated explicitly when used in simulations.

The stimulation was applied as a harmonic force at: (a) a titanium implant ⌀3 × 5 mm screwed into the skull bone, or (b) an acrylic plate ⌀15 × 1.5 mm simulating the interface part of a B71 transducer (Radioear Corp., PA, USA). The implant (screw) was positioned at the standard bone-anchored hearing aid (BAHA) location, (Tjellstrom et al., 2001), approximately 55 mm behind the ear canal opening in line with the upper edge of the pinna, and the B71 transducer plate was placed flat on the mastoid skin 35 mm directly posterior to the ear canal opening (Figure 1B). The 5.4 N static force used to hold the B71 transducer in place was modeled by reducing the density and increasing Young's modulus of the soft tissue directly under the plate according to the method described in (Chang & Stenfelt, 2019). No additional reduction of the skin thickness was conducted. Harmonic force of 1 N magnitude was equally distributed over the face of the stimulation devices perpendicular to the surface. The simulations were carried out in the frequency range of 100–10,000 Hz with a resolution of 25 Hz for 100–500 Hz, 50 Hz for 550–1,000 Hz, and 200 Hz at frequencies above 1,200 Hz resulting in a total of 72 frequencies.

### Cochlear Promontory Vibration

The promontory vibration from the simulations is expressed as three-dimensional (3D) and one-dimensional (1D) accelerance (acceleration divided by stimulating force). The 3D accelerance is calculated as the square root of the sum of squares of accelerance spatial components (Stenfelt & Goode, 2005b). The 1D accelerance is the medial accelerance component (*x* component).

### Experiments’ Overview

Despite the abundance of cochlear promontory measurements in the literature, only a relatively small number is presented in this work. The criterion for inclusion in this study is a stimulation force that was either directly measured or could be well approximated by the stimulation techniques used in the studies. This meant that only the experiments that either reported vibration normalized by the force from a BC transducer or reported both cochlear promontory vibrations and the output force from the BC transducer (either directly or as a driving voltage-force transfer function) were included.

Implant stimulation is applied by a 3 mm screw (Stenfelt & Goode, 2005b), a 4 mm titanium fixture (Eeg-Olofsson et al., 2008, 2011), a BI300 titanium implant (Cochlear BAS; Molnlycke, Sweden) (Dobrev et al., 2019; Hakansson et al., 2008), a transducer placed in a recess in the temporal bone (Hakansson et al., 2010) or a custom made aluminum implant (Rigato et al., 2018). The implants were located at or near the standard BAHA location, except in two studies (Hakansson et al., 2010; Rigato et al., 2018) where, although the implant was located behind the ear canal opening, it was closer to the cochlea compared with the standard BAHA location. The stimulation force was applied using a voltage-driven transducer (directly driven) except in one measurement (Hakansson et al., 2008) where the transducer was driven by a sound pressure (acoustically driven).

On-skin stimulation is applied either by the B71 transducer (Eeg-Olofsson et al., 2013) or BAHA Cordelle II transducer (Cochlear BAS; Molnlycke, Sweden) (Dobrev et al., 2016). In both experiments, voltage-driven transducers were located on the surface of the mastoid skin behind the pinna and held in place by a metal headband providing a static force of approximately 5 N.

The cochlear promontory vibration is reported as vibration level (velocity or acceleration) normalized by force from the transducer except in three studies. Hakansson et al. (2008) reported cochlear promontory vibration related to airborne sound pressure level, and two studies (Dobrev et al., 2019; Hakansson et al., 2010) reported the cochlear promontory vibration in relation to the voltage to the transducer. The stimulation force in these studies were obtained using the transducers’ transfer functions reported in the studies, that is, the output force in relation to sound pressure level or input voltage. To facilitate comparison among the different studies, all outcome measurements not reported as accelerance were recalculated as accelerance.

The 3D vibration data were obtained by either a triaxial accelerometer (Stenfelt & Goode, 2005b) or a 3D LDV (Dobrev et al., 2019). In all other studies, 1D vibration data were obtained by a 1D LDV in the direction approximately matching the medial axis.

## Results

**Figure 2.**
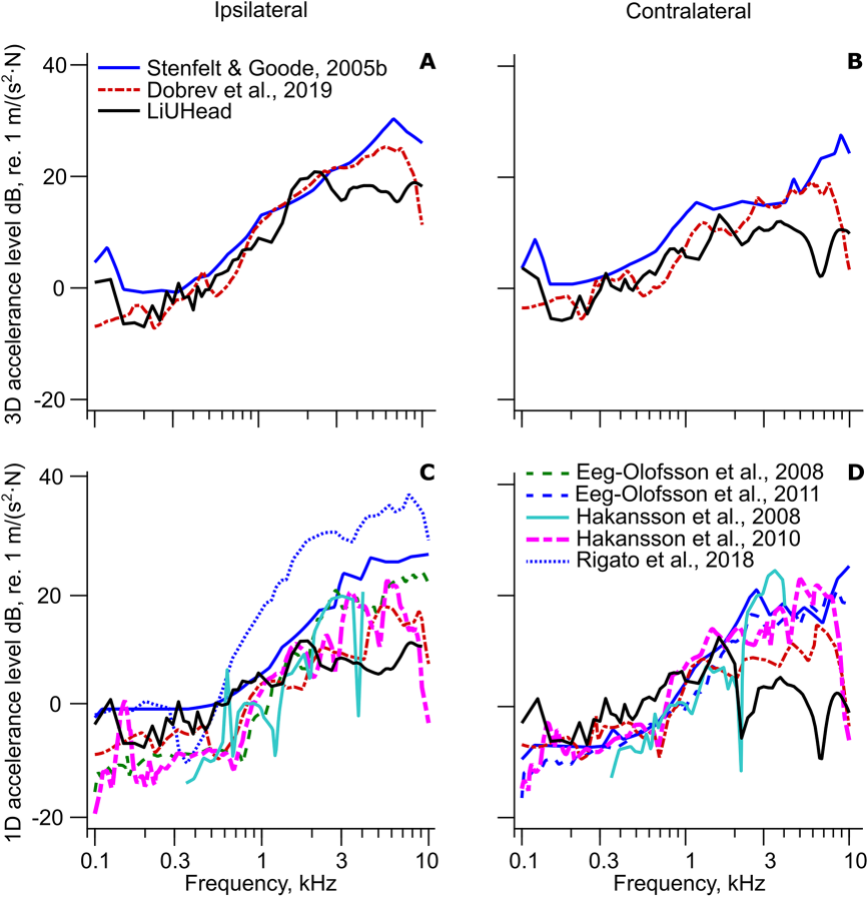
Implant Stimulated Cochlear Promontory Accelerance Levels. Cochlear promontory accelerance levels based on 3D data with (A) ipsilateral and (B) contralateral stimulation and based on 1D data with (C) ipsilateral and (D) contralateral stimulation. References to experiments are provided in the legend, and each line type corresponds to a single study. The LiUHead legend entry indicates the LiUHead standard model with implant stimulation.

**Figure 3.**
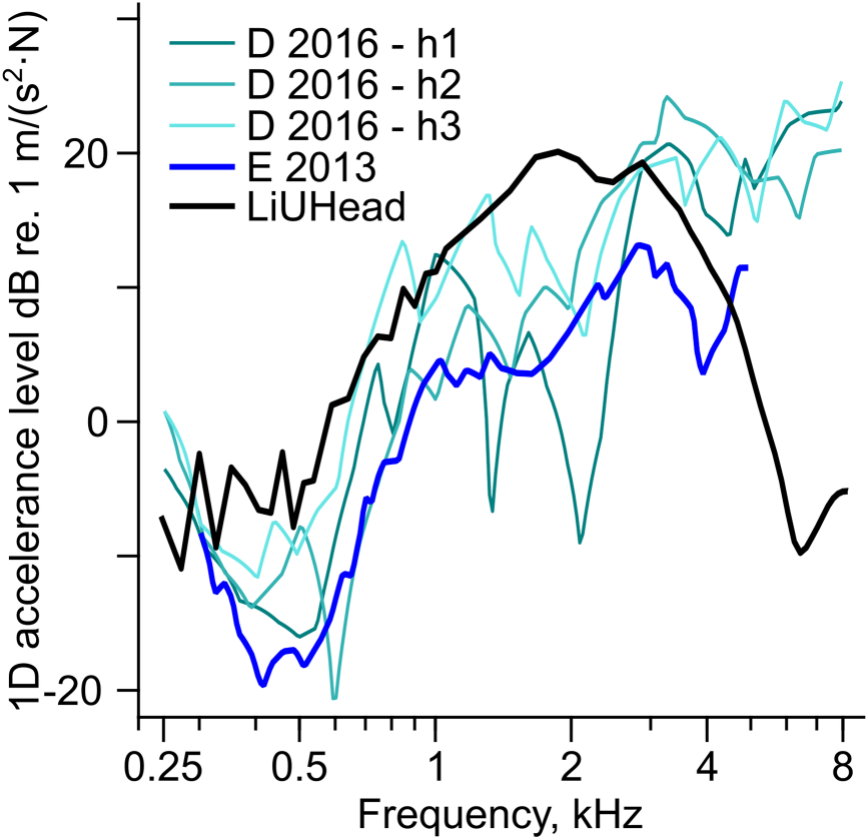
On-Skin-Stimulated Cochlear Promontory Accelerance Levels. The ipsilateral 1D cochlear promontory accelerance with stimulation on the skin. Legend: E 2013—(Eeg-Olofsson et al., 2013), D 2016 h1, h2 and h3—(Dobrev et al., 2016) heads 1, 2, and 3 respectively, and LiUHead—LiUHead standard model with on-skin stimulation.

**Figure 4.**
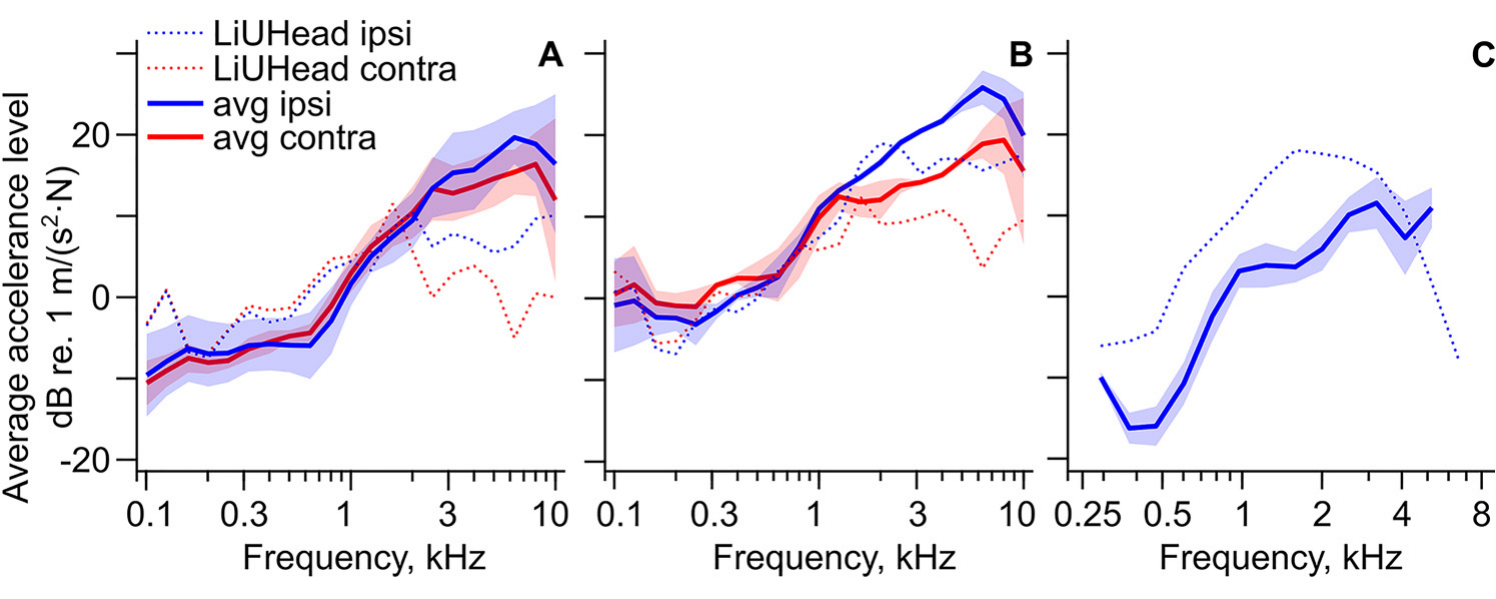
Average Cochlear Promontory Accelerance Levels. The average cochlear promontory accelerance levels from the studies with (A) implant stimulated 1D data, (B) implant stimulated 3D data, and (C) on-skin stimulated 1D data. Experiments in panel A: ipsilateral and contralateral (Dobrev et al., 2019; Hakansson et al., 2010; Stenfelt & Goode, 2005b), ipsilateral only (Eeg-Olofsson et al., 2008), contralateral only (Eeg-Olofsson et al., 2011). Experiments in panel B: (Dobrev et al., 2019; Stenfelt & Goode, 2005b). Experiments in panel C: (Dobrev et al., 2016; Eeg-Olofsson et al., 2013).

**Figure 6.**
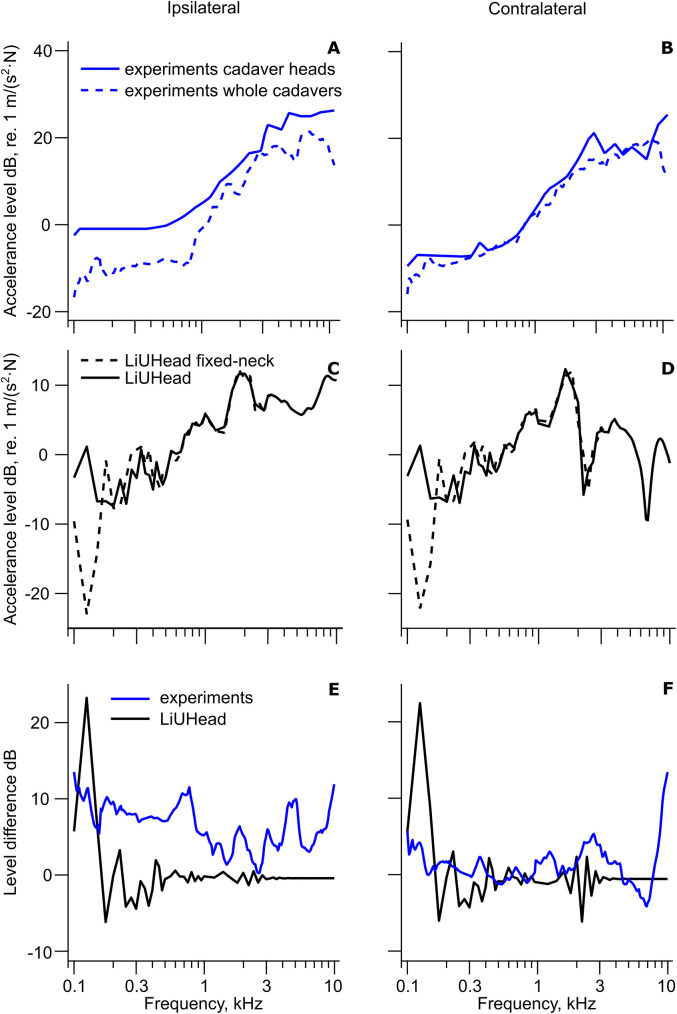
Cadaver Head and Whole Cadaver Cochlear 1D Promontory Accelerance Levels as a Function of Implant Stimulation. Average cochlear 1D promontory accelerance levels from experiments using cadaver heads and whole cadavers with (A) ipsilateral and (B) contralateral implant stimulation. Cadaver head experiments: (Stenfelt & Goode, 2005b) ipsilateral and contralateral. Experiments with whole cadavers (Hakansson et al., 2010) ipsilateral and contralateral, (Eeg-Olofsson et al., 2008) ipsilateral only, (Eeg-Olofsson et al., 2011) contralateral only. Simulation results with the LiUHead standard model and the fixed-neck model with (C) ipsilateral and (D) contralateral implant stimulation. The difference between cadaver head and whole cadaver results and between LiUHead standard and fixed-neck models with (E) ipsilateral and (F) contralateral implant stimulation.

**Figure 7.**
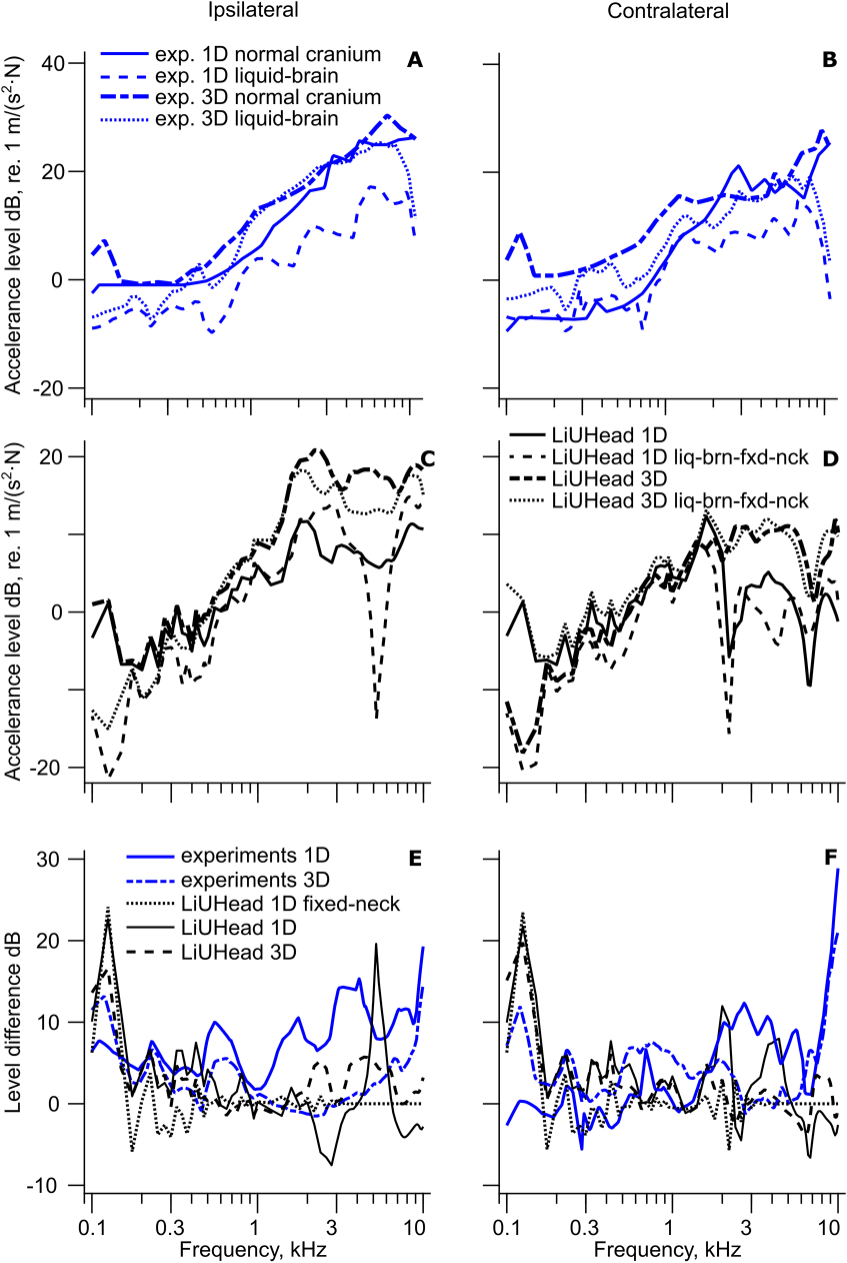
Effect of Implant Stimulation With Cadaver Head Alterations: Effects of Replacing the Brain With Liquid. Cochlear promontory accelerance with implant stimulation from cadaver head experiments with normal cranial content and liquid-filled cranium (A) ipsilateral and (B) contralateral. Normal cranium content data are from Stenfelt and Goode (2005b), and liquid-filled cranium data are from Dobrev et al. (2019). Simulation results (C) ipsilateral and (D) contralateral with the LiUHead standard model and the liquid-brain-fixed-neck model. The difference between experiments with normal cranial content and liquid-filled cranium, and the LiUHead standard and the liquid-brain-fixed-neck models, (E) ipsilateral and (F) contralateral. Panels (E) and (F) include the fixed-neck simulation data from Figure 6E and F labeled “LiUHead 1D fixed-neck”.

**Figure 8.**
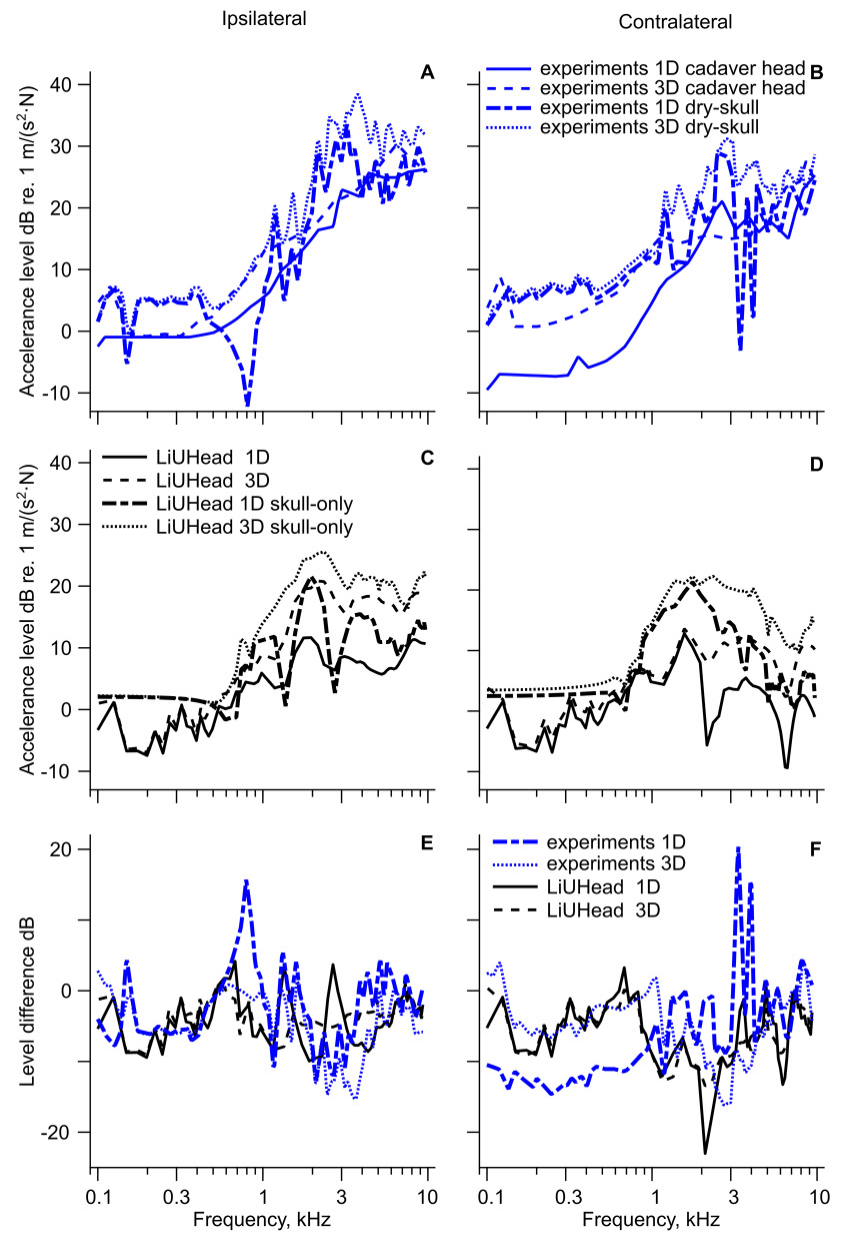
Cadaver Head and Dry Skull Cochlear Promontory Accelerance as a Function of Implant Stimulation. Cochlear promontory accelerance with implant stimulation from severed cadaver heads and dry skulls (A) ipsilateral and (B) contralateral. Cadaver head data are from Stenfelt and Goode (2005b), and dry-skull data are from Stenfelt et al. (2000). Simulation results (C) ipsilateral and (D) contralateral promontory accelerance with LiUHead standard model and skull-only model. The difference between experiments with whole cadaver head and dry-skull and between LiUHead standard and skull-only models (E) ipsilateral and (F) contralateral.

A compilation of the 3D and 1D cochlear promontory accelerance with implant stimulation in the skull bone is shown in Figure 2 including the predictions from the LiUHead standard model. The cochlear promontory accelerance with skin stimulation is assessed and compared with the LiUHead standard model in Figure 3, while Figure 4 summarizes all cochlear promontory accelerances as average and standard deviation (SD). Results with the LiUHead model with head manipulations are compared with the experiments in Figures 6 and 7. Finally, BC vibration through a skull without interior and soft tissue is assessed in Figure 8.

1D = one-dimensional; 3D = three-dimensional.

1D = one-dimensional.

1D = one-dimensional; LiUHead ipsi = LiUHead standard model ipsilateral data; LiUHead contra = LiUHead standard model contralateral data; avg ipsi = averaged ipsilateral experiments; avg contra = averaged contralateral experiments.

*Note.* Shaded areas indicate standard deviations. All values are averaged over third octave frequency bands.

1D = one-dimensional.

1D = one-dimensional.

### Compilation of Cochlear Promontory Accelerances

The experimental cochlear promontory accelerances with stimulation at the BAHA implant in the skull are presented and compared with the LiUHead standard model simulations in Figure 2. The experimental root-mean-squared (RMS) 3D accelerances at the ipsilateral cochlear promontory (Figure 2A) show a general increase with a frequency of around 20 dB/decade between 0.3 and 7 kHz. The Dobrev et al. (2019) and Stenfelt and Goode (2005b) RMS accelerances are within 5 dB except at the highest and lowest frequencies where the values from Dobrev et al. (2019) are 10 dB below the Stenfelt and Goode (2005b) data. The contralateral RMS accelerances (Figure 2B) show 0–5 dB lower values in the Dobrev et al. (2019) data compared with the Stenfelt and Goode (2005b) at frequencies between 0.15 and 7 kHz with 10–20 dB differences at the very high and low frequencies. The similarity between ipsilateral and contralateral differences indicates that the origin for the differences is in the stimulation or measurement system, and not due to differences in vibration transmission in the skull bone itself. The LiUHead standard model simulations are within 5 dB of the experimental data for both ipsilateral and contralateral stimulation up to 4 kHz above which the simulations are 10–15 dB below the experimental data.

The overall tendency of the ipsilateral experimental 1D accelerances (Figure 2C) is a relatively flat response at frequencies up to 0.6 kHz, above which the accelerances increase with 20–30 dB to 4 kHz. Above 4 kHz, there is again a flatter response where some datasets fall abruptly 10 dB at the highest frequencies. The contralateral experimental 1D accelerances (Figure 2D) also indicate relatively flat responses up to 0.6 kHz above which it increases 20–30 dB up to 2–3 kHz. At frequencies above 2–3 kHz, the contralateral responses are flatter but vary unsystematically 5–10 dB up to 10 kHz. Compared with the experimental 3D data (Figure 2A and B) the 1D data have 0–20 dB lower values.

The inter-study variability of the ipsilateral (Figure 2C) accelerances is 10–25 dB with larger inter-study differences at the higher frequencies. The ipsilateral accelerances reported in the Rigato et al. (2018) study have 10–25 dB greater levels compared with the other studies. They used a specially designed implant that was placed closer to the cochlea compared with the standard BAHA location, which may explain this difference. A stimulation position closer to the cochlea has been shown to improve the vibration at the cochlear promontory, especially at the higher frequencies (Eeg-Olofsson et al., 2008; Stenfelt & Goode, 2005b). The Hakansson et al. (2008) data are from one cadaver head which may explain the greater variability in the result compared with the other studies that report medians or averages from several cadaver heads. If the Rigato et al. (2018) data are ignored, most ipsilateral cochlear promontory accelerances are within 10 dB except at frequencies close to 10 kHz where there are substantial deviations among the studies. The variability in the contralateral cochlear promontory accelerances is less than in the ipsilateral data where the different studies mainly fall within 5–10 dB (Figure 2D).

The LiUHead standard model shows 1D accelerance levels that are in line with the largest accelerance levels at frequencies below 1 kHz and in line with, or 5–10 dB lower than, the lowest levels at frequencies above 3 kHz (ignoring the antiresonances in the contralateral accelerance). When analyzing the accelerances of the cochlear promontory in the LiUHead, the x direction results, which are presented in Figure 2C and D, were around 10 dB below the accelerance levels in the z direction at frequencies above 1.5 kHz. This explains the seemingly better agreement between LiUHead predicted accelerances based on 3D data in Figure 2A and B compared with the 1D data in Figure 2C and D.

Individual cochlear promontory accelerance with on-skin stimulation from three cadaver heads (Dobrev et al., 2016) are shown together with average data from postmastoidectomy live patients (Eeg-Olofsson et al., 2013) in Figure 3. These data are from 1D measurements on the ipsilateral cochlear promontory. Overall, the vibration level decreases 10–20 dB between 0.1 and 0.5 kHz and increases with frequency by 30–40 dB up to 3 kHz except for antiresonances seen in the individual data. At frequencies above 3 kHz, the experimental accelerances show relatively flat responses or even a decline. Compared with the 1D accelerances presented in Figure 2C, the accelerances in the live human heads are on average 5 dB lower than the accelerances in the cadaver heads. The Eeg-Olofsson et al. (2013) data were obtained in a group of postmastoidectomy patients. This means, that in this the group, the stimulation position was posterior to the conventional B71 stimulation position. Such alteration of stimulation position reduces the sensitivity of BC hearing and the vibration of the ipsilateral cochlear promontory by a couple of decibels (Eeg-Olofsson et al., 2008; Stenfelt, 2012b). But at the same time, the mastoidectomy itself is predicted to improve the high-frequency ipsilateral cochlear promontory vibration (Prodanovic & Stenfelt, 2020). Moreover, the 1D vibration data in Eeg-Olofsson et al. (2013) were measured by the LDV through the mastoidectomy which is in the medial-posterior direction (x-y direction), which is slightly different from vibration measurement directions through the ear canal.

The simulated accelerance from the LiUHead is in the upper range of the individual cadaver head measurements up to 3 kHz. At higher frequencies, the simulated accelerance drops significantly compared with the experimental data and is about 25 dB below the experimentally obtained accelerances at frequencies above 6 kHz. One possible explanation for the high-frequency attenuation in the LiUHead data is the skin thickness in the mastoid region of the model that ranges from 10 mm to about 13 mm. That is significantly thicker than in an average human, and due to the greater compliance of the thicker skin, it results in greater reduction of high-frequency vibration transmission. However, the parameter value alterations of the soft tissues to mimic the 5.4 N static force implemented here, improves the BC transmission through the soft tissues by around 5 dB at the high frequencies compared with the nonaltered soft tissue of the LiUHead. This is in line with experimental results on static force variations (Toll et al., 2011).

The averages of the experimentally obtained accelerances are presented in Figure 4 along with the predictions by the LiUHead standard model. Plus and minus one SD around the mean is indicated by the shaded areas. The means are computed as weighted averages over the studies using the number of ears in each study as the weight

(1) $$\mathrm{AVG}=\left(\sum_{i=1}^{n}w_{i}X_{i}\right)/\left(\sum_{i=1}^{n}w_{i}\right),$$

Where AVG is the mean value, *w* is the number of ears in a study, *X* is the accelerance from the same study and *i* is the summation index ranging from 1 to *n*, *n* being the total number of studies used in averaging. The SD is calculated as the square root of the weighted variance

(2) $$SD=\sqrt{\left(\sum_{i=1}^{n}w_{i}{\left(X_{i}-AVG\right)}^{2}\right)/\left(\sum_{i=1}^{n}w_{i}\right)}.$$

The results with the acoustically driven transducer (Hakansson et al., 2008) and the results from the study with custom implant (Rigato et al., 2018) were not included in the averaging. The former study is excluded due to large harmonic distortion reported at low and high frequencies, and the latter because the reported values are approximately 10 dB larger compared with the cluster of values from other studies, owning to the fact that the implant was located much closer to the inner ear compared with the standard BAHA location. The 1D accelerances (Figure 4A) of the LiUHead model show 0–5 dB higher values compared with the experimental averages at frequencies up to 2 kHz and at frequencies below 3 kHz, the accelerances from the LiUHead standard model are mostly within the experiments’ SD. At frequencies above 3 kHz, the simulated 1D accelerances are mostly flat with frequency (ipsilateral) or decreases slightly with frequency (contralateral) and are 10–15 dB below the experimental averages. The averaged experimental 3D accelerances in Figure 4B show a similar trend as the 1D experimental averages in Figure 4A but with 5–10 dB higher values. Here, the simulation data are more consistent with the experiments and are within a few decibels from the averages up to 3 kHz, above which the simulated accelerances are approximately 10 dB below the average experimental accelerances. The simulated cochlear promontory accelerance from the LiUHead standard model with skin stimulation is shown together with the experimental averages in Figure 4C. Here, the experimental levels increase more irregularly with frequency with a relatively flat portion between 1.5 and 3 kHz. The LiUHead accelerance peaks at 2 kHz with 5–10 dB greater levels compared with the experimental average at frequencies below 3 kHz. The averages and SDs from Figure 4 are presented in Table 2.

**Table 2.** Cochlear Promontory Accelerance From the Experiments—Average Values and Standard Deviation.

1D cochlear promontory data with BC implant stimulation																					
Frequency, kHz	0.1	0.125	0.16	0.2	0.25	0.315	0.4	0.5	0.63	0.8	1	1.25	1.6	2	2.5	3.15	4	5	6.3	8	10
Ipsi AVG	−10.4	−8.7	−7.4	−7.5	−7.3	−6.5	−6.4	−6.3	−6.4	−3.8	1.2	5.3	8.2	10.3	15.0	16.7	16.9	18.5	21.0	20.7	18.9
Ipsi SD	5.0	4.3	4.3	4.0	3.5	3.3	3.3	3.3	4.0	4.3	3.0	2.0	3.0	3.2	3.5	4.6	4.5	3.8	3.0	4.5	8.0
Contra AVG	−11.2	−9.6	−8.1	−8.5	−8.0	−6.6	−5.8	−4.8	−4.0	−0.9	2.8	6.0	8.9	11.4	14.8	14.0	14.9	15.8	16.7	18.1	14.7
Contra SD	2.7	2.1	1.8	1.5	0.9	1.3	1.4	0.7	1.1	1.6	2.4	2.8	2.0	2.9	3.6	3.1	3.1	3.1	2.5	3.7	9.4
**3D cochlear promontory data with BC implant stimulation**																					
Frequency, kHz	0.1	0.125	0.16	0.2	0.25	0.315	0.4	0.5	0.63	0.8	1	1.25	1.6	2	2.5	3.15	4	5	6.3	8	10
Ipsi AVG	−0.2	0.4	−2.1	−2.2	−3.0	−1.4	0.6	1.6	3.1	7.0	11.7	14.0	15.7	17.5	20.1	21.6	22.9	25.3	27.4	25.9	21.5
Ipsi SD	6.0	5.7	2.3	1.7	2.7	1.0	0.7	1.2	2.7	1.5	0.5	0.2	0.4	0.6	0.5	0.2	0.6	1.1	2.2	2.6	5.5
Contra AVG	1.1	2.4	−0.3	−0.7	−0.7	1.9	2.8	2.9	3.4	6.6	10.8	13.3	12.7	13.0	14.7	15.0	16.0	18.0	20.1	20.8	17.3
Contra SD	4.2	4.9	1.6	1.6	2.3	0.4	0.7	2.2	3.4	3.5	2.9	1.8	2.0	2.5	1.0	0.2	0.2	0.4	1.9	4.3	9.3
**1D cochlear promontory data with on-skin stimulation**																					
Frequency, kHz	0.1	0.125	0.16	0.2	0.25	0.315	0.4	0.5	0.63	0.8	1	1.25	1.6	2	2.5	3.15	4	5	6.3	8	10
Ipsi AVG	−4.1	−3.3	−2.5	−2.3	−3.8	−9.9	−16.6	−16.3	−10.7	−2.0	3.9	4.7	4.5	6.8	11.3	12.8	8.3	12.2	12.2	9.4	−2.6
Ipsi SD	1.9	1.8	1.6	1.5	1.3	0.6	2.0	2.6	2.8	3.2	2.2	2.9	2.0	2.8	2.3	3.4	4.8	2.6	3.8	6.1	13.0

*Note.* The data are the same as presented in Figure 4. The top and middle parts of the table show 1D and 3D accelerance, respectively, with BC implant stimulation, and the bottom part shows the on-skin stimulation accelerance. Units of the displayed accelerances are dB re. 1 m/(s^2^·N). The frequencies correspond to third-octave band frequencies.

1D = one-dimensional; 3D = three-dimensional; BC = bone conduction; Ipsi = ipsilateral, Contra = contralateral; AVG = averages calculated according to equations (1) and (2); SD = standard deviations calculated according to equations (1) and (2).

### Simulations of Manipulation of Intracranial Content

**Figure 5.**
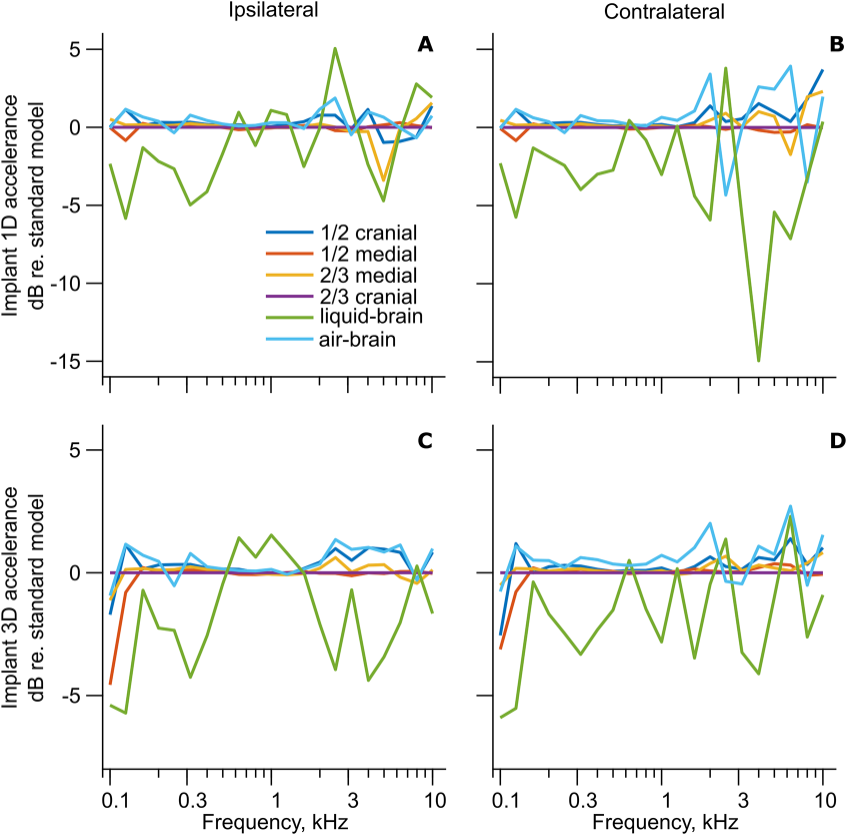
Effects of Cranial Content Manipulation With Implant Stimulation on Simulation Results. A third-octave-averaged cochlear promontory accelerance level with head manipulations models relative to the LiUHead standard model. (A) Ipsilateral 1D differences, (B) contralateral 1D differences, (C) 3D ipsilateral differences, and (D) 3D contralateral differences.

The effect of cranial content on the 1D and 3D cochlear promontory accelerances, based on simulations with the LiUHead using stimulation at the BAHA position, is shown in Figure 5. Four of the simulations explored the predicted effect of brain and intracranial fluid leakage by reductions of the brain and CSF to 2/3 or 1/2 of the height along either medial or cranial axis (Figure 1D to G). One extreme simulation is when the entire content in the cranial space is replaced by air (air-brain) and the last simulation is when the brain is replaced by CSF-like liquid (liquid-brain). Figure 5 shows that replacing part of the cerebral volume with air results in nearly no change of cochlear promontory vibrations relative to the standard model on either the ipsilateral or contralateral side of the head, for both 1D and 3D. The maximum difference at a few frequencies was less than 5 dB, but for most frequencies, the difference was below 1 dB. The same behavior is found when the results with the air-brain model are compared with the standard model; the changes are limited to 2 dB, but at most frequencies, the changes are close to 0 dB. Only the liquid-brain model resulted in noticeable changes that were up to 5 dB for 3D accelerance and up to 15 dB for 1D accelerance. The changes with liquid-brain were mostly negative except between 1 and 3 kHz on the ipsilateral side where the liquid-brain manipulation increased the cochlear promontory vibration by approximately 1 dB for 3D and up to 5 dB for 1D accelerances. Consequently, modeling of the replacement of the cranial content by liquid seems to overall reduce the cochlear promontory vibration when the stimulation is at the BAHA position. Because of the small differences found in Figure 5 between the standard model and the models with brain and CSF volume replaced by air, no further analysis of these conditions is conducted. However, the effects of replacing the cranial content with liquid (liquid-brain manipulation) will be analyzed in relation to experimental data.

1D = one-dimensional; 3D = three-dimensional; 1/2 cranial = cerebrum reduced to 1/2 height along cranial axis; 1/2 medial = cerebrum reduced to 1/2 width along medial axis; 2/3 medial = cerebrum reduced to 2/3 width along medial axis; 2/3 cranial = cerebrum reduced to 2/3 height along cranial axis; liquid-brain = liquid brain model; air-brain = air brain model.

### Effects of Severing the Head

The severed 1D cadaver head data in Figure 6A and B are the accelerances from one study (Stenfelt & Goode, 2005b) that used cadaver heads with stimulation close to the BAHA stimulation position. The other study that used the standard BC implant for stimulation on cadaver heads near the BAHA location (Dobrev et al., 2019) also flushed the intracranial content with fluid and thereby replaced the brain with liquid. This experimental condition was decided to be investigated with the liquid-brain simulations. The whole cadaver data are from three studies where the ipsilateral data are averaged from Eeg-Olofsson et al. (2008) and Hakansson et al. (2010), and the contralateral data are averaged from Eeg-Olofsson et al. (2011) and Hakansson et al. (2010). The simulated 1D accelerances with the LiUHead standard model and the fixed-neck model are shown in Figure 6C and D. The differences between the accelerances (in dB) between severed cadaver heads and whole cadavers as well as the differences between the simulated accelerances with the standard and fixed-neck models are shown in Figure 6E and F. When analyzing these comparisons, the simulated ipsilateral and contralateral cochlear promontory accelerances show an increase in cochlear promontory vibration level for frequencies below 200 Hz when the neck boundary is free compared with when it is constrained. In the experimental data, the severed cadaver heads give 5–10 dB greater ipsilateral cochlear promontory vibration levels at frequencies below 1 kHz and in the range of 0–8 dB at frequencies above 1 kHz compared with the whole cadaver cochlear promontory vibration levels. At the contralateral cochlear promontory, no clear trend is visible, but the severed cadaver heads result in slightly greater cochlear promontory vibration levels compared with the whole cadaver data at the lowest and highest frequencies investigated. One explanation for the observed discrepancy between the experimental ipsilateral and contralateral results when comparing severed and whole cadavers is that the stimulation position in Stenfelt and Goode (2005b) used for this comparison was slightly closer (approximately 40 mm from the ear canal opening) than the standard BAHA position (55 mm behind the ear canal opening) used in the whole cadaver data. A closer stimulation position to the inner ear improves ipsilateral but not contralateral cochlear promontory results (Eeg-Olofsson et al., 2008, 2011). Even so, there seems to be a region at the lowest frequencies, below 200 Hz, that suggests better cochlear promontory vibration levels at both the ipsilateral and contralateral sides in the experimental data that is also seen in the simulated data. Consequently, these comparisons and simulations indicate that severing the head leads to improved vibration levels of the skull with BC excitation at frequencies below 200 Hz.

### Effects of Replacing Intracranial Content With Fluid

The effect of replacing the skull interior with CSF-like liquid is explored in Figure 7. In the experiment where the skull interior was replaced by fluid (Dobrev et al., 2019), the neck region of the head was fixed by a rod through the vertebrae. The top row in Figure 7 (Figure 7A and B) shows 1D and 3D cochlear promontory accelerance from the cadaver head experiments using implant stimulation and fluid-filled cranium (Dobrev et al., 2019). The other dataset in Figure 7A and B with 1D and 3D cochlear promontory accelerance has the normal cranial content and is from Stenfelt and Goode (2005b). The corresponding simulations from the LiUHead are displayed in the middle row (Figure 7C and D) with 1D and 3D accelerance from simulations with the standard model mimicking Stenfelt and Goode (2005b), and simulations with the liquid-brain-fixed-neck model, mimicking Dobrev et al. (2019) data. The bottom row (Figure 7E and F) shows the 1D and 3D accelerance difference between having a fluid-filled cranium and fixed neck, and normal cranial content and free neck. In addition, to facilitate separating the effects of fixed-neck and liquid-brain, the 1D simulated difference between the standard model and the fixed-neck model from Figure 6E and F is also included in Figure 7E and F.

On the ipsilateral side (left column) the experimental differences (1D and 3D) decrease nonmonotonically from around 10 dB at 100 Hz to 0 dB at 1 kHz and increase to 15–20 dB at 10 kHz, indicating that replacing the brain with liquid and constraining the neck decreases the cochlear accelerance at low and high frequencies. The decrease is more noticeable in the 1D than in the 3D data, especially at frequencies above 1 kHz where there is a 15 dB difference between 1D and 3D results at 4 kHz. A similar trend is observed on the contralateral side, but the 1D and 3D differences are more similar and within 0–10 dB for almost the entire frequency range. The exception is at the highest frequencies, above 7 kHz, where the differences increase to a maximum of 30 dB. The simulated difference between the standard model and the liquid-brain-fixed-neck model follows the general trend of the experimental data at frequencies below 2 kHz. At higher frequencies, the simulation-based differences remain within 5 dB whereas the experimental differences increase. There is an overall similarity between the simulations and experimental results where the simulations with the liquid-brain-fixed-neck model showed 5–15 dB lower promontory accelerance compared with the standard model.

Finally, panels E and F include the difference from Figure 6E and F with simulations with the fixed-neck model. This difference captures the alterations caused by constraining the neck but not replacing the cranial content with fluid. This simulated difference, in 1D, is similar to the other ipsilateral simulations at frequencies below 2 kHz and the contralateral simulations for the entire frequency range. At frequencies above 2 kHz on the ipsilateral side, the simulations using the liquid-brain model show variable results, but there are indications of a lower cochlear promontory vibration at frequencies above 3 kHz with the liquid-brain model compared with normal intracranial content from both simulations (standard model) and experimental data. The overall similarities of the differences between the standard model and the two models with liquid brain (with or without fixed neck) indicate that the effect of the fixed neck was stronger on the cochlear promontory vibration than replacing the brain with fluid.

### Simulation of the Skull Without Soft Tissue and Intracranial Content

The cochlear promontory accelerances with BAHA stimulation in cadaver heads (Stenfelt & Goode, 2005b) and from a dry-skull experiment (Stenfelt et al., 2000) are shown in Figure 8A and B as 1D and 3D data. A similar setup is done with the LiUHead and simulations using the standard model and the skull-only model are shown in Figure 8C and D. The difference between the standard model and the skull-only model as well as between cadaver head experiments and dry-skull measurements are shown in Figure 8E and F.

The dry-skull experiments show around 5 dB greater ipsilateral accelerances than the cadaver heads at frequencies below 500 Hz where the responses are relatively flat. The dry-skull accelerances are based on a single skull and show a nonsmooth curve from resonances and antiresonances, which are averaged out in the cadaver heads curves. With that in mind, there are overall greater ipsilateral accelerance levels for the dry-skull data compared with the cadaver heads, even if the accelerances overlap at frequencies of 4 kHz and above. On the ipsilateral side, the dry-skull vibrations show a peak at 3–4 kHz, while the cadaver head data flatten out at frequencies above 4–6 kHz without a distinct peak. On the contralateral side, the cadaver head 1D accelerance levels are 10–15 dB below the others at frequencies below 1.5 kHz. At higher frequencies the results vary with sharp peaks and valleys, but there is an overall tendency of higher accelerance levels in the dry-skull responses compared with the cadaver head responses.

The skull-only model exhibits a flat response below 700 Hz with similar levels on both ipsilateral and contralateral sides indicating a rigid body motion. At higher frequencies, the vibration level increases, and peaks at about 2 kHz. The peak values are 20 and 25 dB re. 1 m/(s^2^·N), with a higher and sharper peak on the ipsilateral side. This is more prominent in the 3D case whereas the 1D vibration show consecutive peaks and troughs on the ipsilateral side caused by a series of resonances. The standard model response follows the general shape of the frequency response of the skull-only model but is about 10 dB lower but with a nonflat low-frequency vibration.

Panel E illustrates that the ipsilateral accelerance differences between an air-filled skull without soft tissues and a normal head, either as experiments or simulations, are less than 10 dB for most frequencies. Larger differences occur sporadically at narrow frequency ranges, and the simulations and experimental data are mostly similar. Overall, the accelerances are negative and hover around −5 dB, indicating that the ipsilateral response in an “empty skull” (dry skull experiment or skull-only model) is around 5 dB higher compared with an intact head. At the contralateral side (Figure 8F), there is a clear difference between 1D experiments and simulations at low frequencies. Below 1 kHz the 1D experimental data are between −15 and −10 dB while all the simulated data and 3D experiments fall mostly in the −5 to 0 dB range. This implies that for the experiments, a dry skull improves the 1D contralateral low-frequency accelerance by up to 15 dB while the simulations predict the contralateral low-frequency improvement from removing the soft tissues and intracranial content to be approximately 5 dB, similar to the ipsilateral simulations data. At higher frequencies, large variations occur but, except for some extreme values at a few frequencies, the improvement from removing soft tissues and intracranial content is between 0 and 10 dB (−10 to 0 dB in Figure 8F). Here the simulations and the experimental data show similar values.

### Transcranial Attenuation

**Figure 9.**
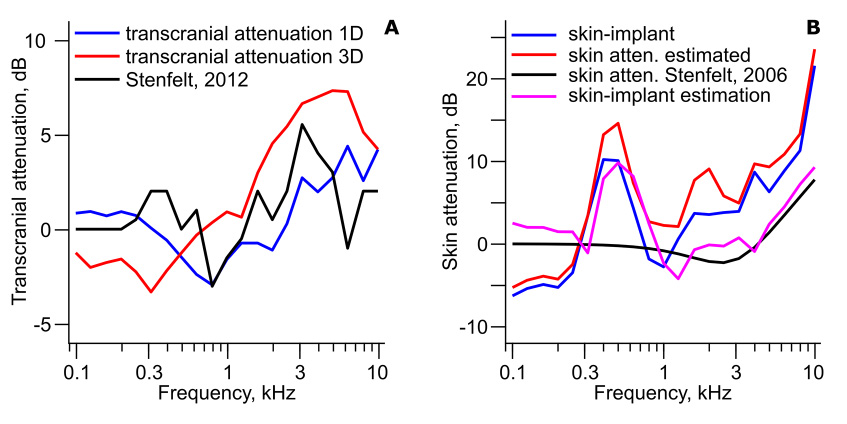
Transcranial Attenuation and Effect of Skin Layer. (A) Transcranial attenuation with implant stimulation from Table 2 data with 1D accelerance levels (blue line) and 3D accelerance levels (red line). The black line is the perceptual estimated transcranial attenuation with stimulation at the implant position from Stenfelt (2012b). (B) Estimations of the skin attenuation from Table 2 (blue line) and when adjusted for stimulation position difference (red line). Also included is the model-based skin attenuation from Stenfelt (2006) (black line) and when the model-based skin attenuation incorporated calibration bias (magenta line).

The transcranial attenuation can be estimated from the experimental data provided in Figure 4 and Table 2. For implant stimulation, ipsilateral and contralateral 1D and 3D cochlear promontory vibration levels are provided. The transcranial attenuation is then computed as the ipsilateral minus the contralateral dB values. The estimated transcranial attenuation based on this computation is shown in Figure 9A for both 1D and 3D data. The 3D data indicate 3–4 dB overall higher attenuation compared with the 1D data where the 1D transcranial attenuation is negative (better contralateral stimulation) at frequencies between 300 and 2.5 kHz. At the highest frequencies, the transcranial attenuation based on the 1D data is around 4 dB. The 3D data transcranial attenuation is negative at frequencies below 800 Hz and increases with frequency up to 7 dB at 6.3 kHz. The 3D transcranial attenuation is reduced to 4 dB at 10 kHz. Also included in Figure 9A is the transcranial attenuation obtained in humans by hearing thresholds in Stenfelt (2012b). This is one of the few studies that have estimated the transcranial attenuation subjectively with the stimulation at the implant position. The subjective transcranial attenuation falls for most part between the estimates from the 1D and 3D data, indicating that the 1D data slightly underestimate the transcranial attenuation while the 3D data slightly overestimate the transcranial attenuation. It should be noted that the subjective transcranial attenuation is within a few decibels from either of the vibration-based estimates of the transcranial attenuation.

1D = one-dimensional; 3D = three-dimensional.

## Discussion

### The Model and the Study

The current study aims to review and compare available experimental data and to provide an assessment of the effects of the experimental protocol and methods used. To this end, simulations in the LiUHead mimicking different set-ups and measurement methods were used to investigate the similarities and differences found between the experimental studies. The advantage of using the LiUHead is that numerical simulations provide a convenient tool to explore a variety of effects caused by head manipulations and stimulation protocols.

One limitation of the study is that not all BC-stimulated cochlear-promontory-vibration experiments were included. The reason is that the reported data could not be recalculated to a comparable stimulation level, presented here as vibration level per force from the transducer. Because of this, such data could not be compared with other experiments. For example, studies by Mattingly et al. (Mattingly et al., 2015, 2020) referenced the cochlear promontory vibrations to the electric voltage applied to a BC transducer but without providing the transducer transfer function (relating voltage applied to the transducer to the output force from the transducer). Consequently, not providing the details of the excitation makes it impossible to make a comparison between studies as well to include them in the current study. As a result, the current study is limited to nine studies (Table 1) where the full stimulations data and cochlear promontory vibration were provided. These studies comprise measurements using cadaver heads, whole cadavers, and live subjects and applied the BC stimulation at both the implant in the skull and on the skin. These studies covered a broad range of experimental conditions, which resulted in using just one or two studies to represent a particular condition.

**Table 1.** Overview of the Experiments Reviewed in this Article.

Study	Live subjects	Whole cadaver	Thawed head	Thiel fixed head	Number of ears	Stimulation type	Measurement type	Measurement axis	Data type
Eeg-Olofsson et al., 2013	**x**				16	1	1D	EC^1^	Median
Eeg-Olofsson et al., 2008		**x**			14	2	1D	EC^2^	Median
Eeg-Olofsson et al., 2011		**x**			14	2	1D	EC^2^	Median
Hakansson et al., 2008		**x**			1	2	1D	EC^2^	Individual data
Hakansson et al., 2010		**x**			3	3	1D	EC^2^	Individual data
Stenfelt and Goode, 2005b			**x**		6	2	1D/3D	EC^2^	Average
								xyz^1^	
Rigato et al., 2018			**x**		8	4	1D	EC^2^	Average
Dobrev et al., 2016				**x**	3	5	1D	EC^2^	Individual data
Dobrev et al., 2019				**x**	5	2	1D/3D	xyz^2^	Median

*Note.* Live subjects—experiments using live patients. Whole cadaver—experiments using whole embalmed cadavers. Thawed head—experiments using frozen severed cadaver heads defrosted before the measurements. Thiel fixed head—experiments using severed Thiel embalmed cadaver heads. Number of ears—number of individual ears used in the experiments. In some cases, ipsilateral data are obtained from both left and right ears. Stimulation type—indicates type and location of a BC device used for the stimulation: (a) B71 on-skin—B71 transducer placed on the mastoid skin behind the pinna, (b) BAHA implant—BAHA implant located at the standard BAHA location in the skull, (c) BC implant—a capsuled balanced electromagnetic separation transducer located at the flat bottom of a recess made in the temporal bone, (d) BC implant—a custom circular aluminum BC implant located in a temporal bone recess, (e) on-skin—BAHA Cordelle II transducer held by a metal band against the mastoid skin behind the pinna (position 5 in the original work). Measurement type—indicates whether 3D or 1D cochlear promontory vibration is measured. Measurement axis—the axis (direction) the measurements of the cochlear promontory vibrations were taken at: EC^1^: laser aimed through wide ear canal opening in a middle ear common cavity. EC^2^: laser aimed through the bony part of the ear canal.

xyz^1^: triaxial accelerometer was used. Data for all 3 coordinate axes. xyz^2^: 3D LDV was used. Data for all three coordinate axes. Data type—indicates how the data are reported.

1D = one-dimensional; 3D = three-dimensional; BAHA = bone-anchored hearing aid; BC = bone conduction; LDV = laser Doppler vibrometry.

### The Cochlear Promontory Vibration Reference

In a study to provide reference data for implantable middle ear hearing devices, Rosowski et al. (2007) provided an estimate of the average and range of the middle ear transfer function based on data from nine studies. In a similar way, the data provided in Figure 4 and Table 2 can be used as a reference for studies using cochlear promontory vibration data to ensure reasonable results. In cadaver head studies, the details and origins of the heads are often unknown. The knowledge of the estimated result could therefore be used to ensure that the measurements are not impacted by fractures or other abnormalities of the heads. Moreover, a significant deviation from the predicted result could indicate a loose mechanical coupling, a problem with the electrical signal, or an issue with the measurement system.

The vibration reference in Figure 4 and Table 2 is with stimulation at the typical position in the skull bone for a BC hearing aid. Consequently, the provided data can only be used for studies with the stimulation position at a similar place. It was found in the current study that the data provided in Rigato et al. (2018), that had a stimulation position closer to the ipsilateral inner ear, resulted in 10–20 dB greater cochlear promontory vibration levels compared with the studies that applied the stimulation at the conventional implant position. Similar increase in cochlear promontory vibration level with a stimulation position close to the inner ear has been reported before (Eeg-Olofsson et al., 2008; Stenfelt & Goode, 2005b).

The average and SD values in Figure 4 and Table 2 are weighted by the number of ears in each experiment (equations (1) and (2)). For the 1D results (Figure 4A), the ipsilateral accelerances were dominated by Eeg-Olofsson et al. (2008) (14 ears), and the contralateral accelerances were dominated by Eeg-Olofsson et al. (2011) (14 ears). The number of ears in these two studies is greater than the number of ears in the other studies combined (Table 1). This dominance resulted in a seemingly low computed SD in Figure 4A compared with the spread of average results in Figure 2C and D. The 3D accelerances (Figure 4B) come from two studies (Dobrev et al., 2019; Stenfelt & Goode, 2005b), which had relatively similar averages, and the corresponding SD is also small. These SD calculations are based on the averages from the included studies, and it can be expected that measurements in a single head can fall outside these values, but averaged accelerances should mainly fall within the provided ranges.

### Effect of the Skin

The data in Figure 4 and Table 2 facilitate investigation of the effect of skin in between the transducer and the skull. This analysis is accomplished by comparing the cochlear promontory vibration levels with stimulation applied on the skin and stimulation applied directly on the skull bone (ipsilateral stimulation, 1D data). Since the stimulation level at both stimulation positions is 1 newton, the difference between the cochlear promontory vibration levels can be used as an estimate of the skin attenuation. This computation is shown as the blue line in Figure 9B. The difference indicates a negative attenuation of around −5 dB at frequencies up to 300 Hz that increases abruptly to around 10 dB at frequencies between 400 and 600 Hz. Above 600 Hz, the attenuation falls to 0 dB at 1 kHz and above this frequency, the attenuation increases with frequency to approximately 20 dB at 10 kHz. Also included in Figure 9B is the theoretical estimation of the skin attenuation (black line) from Stenfelt (2006) using the mechanical point impedance of the skin from Flottorp and Solberg (1976) and the skull impedance from Stenfelt and Goode (2005b). This estimate is 5–10 dB lower than the estimate based on the cochlear promontory vibration at nearly all frequencies above 300 Hz and indicates that a simple mass-spring system cannot explain the effect of the skin in the BC transmission.

One caveat in the estimated effect of the skin is that the stimulation position differs between the skin applied stimulation and the stimulation provided at the implant. According to the cochlear promontory vibration estimates in Eeg-Olofsson et al. (2008) the cochlear promontory vibration level is up to 10 dB higher, but mainly around 5 dB higher, with a stimulation at the mastoid (skin applied stimulation here) compared with a stimulation at the BC implant. Stenfelt (2012b) estimated the average difference between a stimulation at the BC implant position and at the mastoid based on hearing thresholds and reported the mastoid to be 0–5 dB more sensitive. If the data from Stenfelt (2012b) are incorporated in the estimation of the influence from the skin, the effect becomes even greater, which is illustrated with the red line in Figure 9B. Now, the effect of the skin is nearly 15 dB at 400–600 Hz. This low-frequency effect is difficult to explain with the skin attenuation alone. However, one origin of the deviation can be in the estimation of the stimulation force.

For skin-applied stimulation, the estimation of the stimulation force is measured on an artificial mastoid, for example, the Brüel and Kjær type 4,930. The idea is that the force output from the BC transducer should be the same on the artificial mastoid and the human mastoid. In reality, the mechanical point impedance level for a Radioear B71 applied to the artificial mastoid is up to 5 dB higher than at the human mastoid (Flottorp & Solberg, 1976; Surendran & Stenfelt, 2021). This difference affects the resonance frequency of the BC transducer. By using a model of a Radioear B71 transducer, Chang and Stenfelt (2019) estimated the force output on the human mastoid and the artificial mastoid with the same electrical input. This computation indicated, due to a shift in the BC transducer's resonance frequency at around 400 Hz, up to 5 dB higher output levels on the human mastoid compared with the artificial mastoid at frequencies below 400 Hz and around 5 dB lower output levels on the human compared with the artificial mastoid at frequencies above 400 Hz (Surendran & Stenfelt, 2021). A similar computation based on the impedances of a BC implant and the skull simulator used to measure the output force of a BC transducer for skull attachment (Hakansson et al., 2020), indicated 2.5–5 dB higher output levels on the human implant compared with the skull simulator at frequencies below 1 kHz. At frequencies above 1 kHz, the output on the human implant was 2.5 dB lower than that measured on the skull simulator.

The effect of force calibration and the theoretical estimate of skin attenuation from Stenfelt (2006) was combined and is shown as the magenta curve in Figure 9B. This should be compared with the red line that is the difference between skin and implant applied stimulation that has been adjusted for the difference in stimulation positions. It is here apparent that the great impact of the skin at around 500 Hz is not an effect of the skin but is caused by errors that occur during calibrations. At higher frequencies, the theoretical estimate including the effect of calibration is still around 5 dB too low. This could be a result of the calculation of the calibration errors. These errors were based on model computations, and they may have underestimated the effect of the calibration errors at higher frequencies. The difference found can also be a result of an incorrect theoretical model of the skin attenuation used. The resonance frequency for the skin impedance based on the Flottorp and Solberg (1976) parameter values is 3 kHz, and that may be incorrect for vibration transmission through the skin. If this resonance frequency were lowered to 2 kHz, the theory-based estimation would be very similar to the effect of the skin when adjusted for position and calibration. A third possibility for the differences found is that the vibration data are partly from postmortem heads, and the mechanical properties of bone, skin, and soft tissues can differ between living and dead tissue. However, in a study on the sensitivity difference between skin-applied and skull bone-applied BC stimulation based on hearing thresholds (Stenfelt & Hakansson, 1999), the result was very similar to that shown in the blue curve in Figure 9B (Figure 8 in the referenced study). This indicates that the findings in Figure 9B are not due to the use of postmortem heads and that the skin and soft tissue features are similar in live and postmortem heads. It is most likely that the error between the theoretical estimate, and the skin attenuation estimate based on the vibration measurements originates in the calibration of the BC transducer and the theoretical model of skin attenuation.

### Effect of Measurement Method

In most studies, the exact conditions of the cranial contents were not reported. In severed heads, it is likely that fluid from the skull interior leaks out creating an air pocket inside the cranium. This effect was here simulated by a reduction of the volume of the brain and CSF. Total replacement of the brain with liquid simulated experiments where the brain tissue was replaced by fluid.

It was expected that a reduction of the cranial content could influence the cochlear promontory vibration positively since part of the head mass was removed. This was not found, and Figure 5 indicates that the cochlear promontory vibration with stimulation in the mastoid area is independent of the status of the brain. The finding was explained by Stenfelt and Goode (2005b) where it was reported that the average mass of the heads was 3.5 kg, but the loading mass at the stimulation position was only 0.85 kg. They suggested that at frequencies above 100 Hz, the brain and soft tissues are decoupled from the skull vibration and the effect of replacing the intracranial content with air is negligible on the skull bone vibration response. Consequently, replacing the entire brain with air (air-brain) did not influence the cochlear promontory vibration response.

This result is in part corroborated when comparing the results from the dry skull (Stenfelt et al., 2000) and the intact cadaver heads from Stenfelt and Goode (2005b): there are around 5 dB higher accelerance levels in the dry skull compared with the intact cadaver heads (Figure 8). A similar result is found in the simulations of the LiUHead standard model and the skull-only model, the cochlear promontory vibration levels are around 5 dB higher in the skull-only model. This suggests that the soft tissues do have a small impact on the cochlear promontory vibration with implant stimulation. Moreover, the skull bone in the Stenfelt et al. (2000) study was dry, while the skull bones in the Stenfelt and Goode (2005b) were wet. This probably influences the BC wave transmission speed with higher wave speed in a dry bone than in wet bone, but the level differences are similar in the experiments and simulations. The simulations use the same skull bone properties for the LiUHead standard model and the skull-only model, indicating that the dryness of the skull bone does not affect the cochlear promontory vibration level significantly, and the different levels are attributed to the removal of the soft tissues. It should be noted that the vibration response in the Stenfelt et al. (2000) study was obtained at a slightly different position than in the Stenfelt and Goode (2005b) study. Moreover, the dry skull in Stenfelt et al. (2000) had a 5 mm layer of damping material added to the inside of the cranial vault that was not included in the skull-only model here. It is assumed that the damping material attenuates the impact of resonances and antiresonances in the skull bone vibration response but does not affect the overall vibration levels at the cochlear promontory.

Figure 5 reveals an impact from replacing the brain by fluid in the liquid-brain simulations. The effect varies with frequency but gives overall 0–5 dB lower cochlear promontory vibration levels. One explanation for this behavior is that the brain can be decoupled from the skull vibration as it is encapsulated by the CSF. When this space is replaced by homogenous incompressible fluid, there is no vibration decoupling, but the mass of the fluid affects the stimulation at the implant. This hypothesis is supported by an analysis of the mechanical point impedance at the stimulation position. At the lowest frequencies, where the mechanical point impedance at the implant is mass dominated, the implant impedance level computed in the liquid-brain model is around 6 dB higher than in the standard model, indicating a doubling of the loading mass. Also, at frequencies above 1 kHz, where the mass affects the impedance at the implant, the impedance level for the liquid-brain model is 2–3 dB higher than the impedance in the standard model. Consequently, a homogenous liquid in the cranial space reduces the vibration response at the cochlear promontory compared with having a brain surrounded by liquid (CSF).

Another experimental method analyzed is when the neck of the skull is fixed, either by attachment to the body or by a fixation rod in severed heads. This was investigated with the fixed-neck model. This fixation is done over the surface indicated in red in Figure 1C. It is implemented such that all motion is completely constrained over the entire surface: both translational and rotational degrees of freedom are constrained. Neither a head–neck attachment nor the support of a cadaver head during experiments constrains the motion in a way that the fixed-neck boundary condition does. The skull-vertebra connection allows for a triaxial rotation of the head that is constrained by fixed-neck boundary conditions. When a cadaver head is kept in an upright position (Dobrev et al., 2019) the spinal connection was mimicked by a short metal rod and the head was further stabilized around the scalp using a soft band. The fixation in the fixed-neck boundary condition is on the soft tissues, which does not completely constrain motions of the skull bone. Despite differences between experiments and the model for the fixed-neck boundary, the investigation allows analysis of the effects of a head–neck constraint.

In the comparison of the cochlear promontory vibration in severed cadaver heads and full body cadavers, whole cadavers gave 5–10 dB lower levels than the severed cadaver heads on the ipsilateral side at frequencies below 1 kHz. Part of this difference is attributed to differences in the experimental procedures such as differences in the exact stimulation position, the use of accelerometers and LDV for vibration measurements, and that the whole cadavers were embalmed while the severed heads were both embalmed and thawed fresh-frozen. However, both the ipsilateral and contralateral experimental data show an increase in the difference at frequencies below 300 Hz (Figure 6E and F). The simulations indicate an effect on the cochlear promontory vibration with fixing the neck at frequencies below 400 Hz where the greatest effect is at 150 Hz. These results suggest that the effect of severing the heads at the neck affects the vibration response at frequencies below 300 Hz to 400 Hz with significant alterations at frequencies below 200 Hz. This is consistent with a previous report stating that the head–neck attachment is only important at frequencies below 400 Hz (Stalnaker & Fogle, 1971).

### Inter-Study Comparisons

The current study has highlighted the importance of reporting the stimulation in experimental investigations in comparable and replicable ways. One prerequisite to turn a hypothesis into theory is the replications of experiments. When reviewing published data on cochlear promontory vibration, a significant number of studies did not provide sufficient information about the stimulation to enable comparison among studies. This is unfortunate since a greater number of included studies would have allowed more in-depth analyses of method effects as well as providing more robust predictions. In the present study, differences between studies, such as exact stimulation position, type of implant, stability of implant, calibration methods, and the type of headrest, could not be analyzed.

One observation is the relatively large variability between studies at the highest frequencies (e.g., Figure 2). This could be an effect of the coupling between the BC transducer and the implant in the skull. Among the studies, different types of implants were used (Table 1) but also different ways to attach to the implant, for example by screwing the transducer to the implant or by a snap-coupling. It has been shown that the connection between the transducer and the implant/skull introduces a compliance that can affect the high-frequency BC stimulation (Hakansson et al., 2020). Since the impedance of the skull and the skull simulator used for calibration differ, and the connection to attach the BC transducer can be different between the skull and skull simulator, the high-frequency stimulation level can differ between the skull and the skull simulator. These differences can then lead to the relatively large high-frequency deviations in cochlear promontory vibration levels observed between studies.

## Conclusions

Nine published studies of BC stimulated cochlear promontory vibration were compiled and analyzed using the LiUHead finite element model. Experiments with implant stimulation showed overall similar responses with values typically within 10 dB, independent of the exact experimental setup and cadaver/cadaver head preparations. Assessment of studies with on-skin stimulation revealed that cadaver head measurements are consistent with the data from living humans. Comparing on-skin-stimulated results with implant-stimulated results suggests that a simple impedance-based model is not sufficient to capture the attenuation effect from the skin. Calibration errors due to impedance mismatch bias the result by up to 10 dB.

Partial or complete replacement of the brain with air does not affect the cochlear promontory vibration, but replacing the brain with liquid reduces the cochlear vibration level by up to 5 dB. During BC vibration, the brain is vibrationally decoupled from the head, while liquid acts as a mass-load on the head. An intact head–neck connection affects the vibration of the head at frequencies below 300 to 400 Hz with a significant vibration reduction at frequencies below 200 Hz. Removal of all soft tissues and cranial content from the head, thus leaving only the bony skull, increased the overall cochlear promontory vibration level by around 5 dB.
